# Vitamin D, Homocysteine, and Folate in Subcortical Vascular Dementia and Alzheimer Dementia

**DOI:** 10.3389/fnagi.2017.00169

**Published:** 2017-05-30

**Authors:** Rita Moretti, Paola Caruso, Matteo Dal Ben, Corrado Conti, Silvia Gazzin, Claudio Tiribelli

**Affiliations:** ^1^Neurology Clinic, Department of Medical, Surgical and Health Sciences, University of TriesteTrieste, Italy; ^2^Italian Liver Foundation, Centro Studi FegatoTrieste, Italy

**Keywords:** sVAD, AD, homocysteine, vitamin D-OH 25, neurodegeneration, inflammation

## Abstract

Dementia is a worldwide health problem which affects millions of patients; Alzheimer's disease (AD) and subcortical vascular dementia (sVAD) are the two most frequent forms of its presentation. As no definite therapeutic options have been discovered, different risk factors for cognitive impairment have been searched for potential therapies. This report focuses on the possible evidence that vitamin D deficiency and hyper-homocysteinemia can be considered as two important factors for the development or the progression of neurodegenerative or vascular pathologies. To this end, we assessed: the difference in vascular risk factors and vitamin D-OH25 levels among groups of sVAD, AD, and healthy age-matched controls; the association of folate, B12, homocysteine, and vitamin D with sVAD/AD and whether a deficiency of vitamin D and an increment in homocysteine levels may be related to neurodegenerative or vessel damages. The commonly-considered vascular risk factors were collected in 543 patients and compared with those obtained from a healthy old volunteer population. ANOVA group comparison showed that vitamin D deficiency was present in demented cases, as well as low levels of folate and high levels of homocysteine, more pronounced in sVAD cases. The statistical models we employed, with regression models built, and adjustments for biochemical, demographic and neuropsychiatric scores, confirmed the association between the three measures (folate decrease, hyperhomocysteinemia and vitamin D decrease) and dementia, more pronounced in sVAD than in AD.

## Introduction

Dementia is a major clinical condition, which increases in prevalence and incidence, more rapidly with advancing age. The consequence is the macroscopic alteration of the daily living abilities. However, the different types of dementia have various causes and pathogenesis (Reitz et al., 2011). It is accepted that many cerebrovascular alterations coexist or are determinant even in neurodegenerative disorders such as AD, and the two different conditions share many different risk factors (Jellinger, 2013).

Whereas, the operative definition of AD is established, it is more difficult to define the criteria for subcortical vascular dementia, which is a clinical entity related to small vessel muscle cells disease with consequent hypo-perfusion, diffuse ischemic white matter lesions, and incomplete ischemic damage (Rockwood, 2003; Korczyn et al., 2012; Jellinger, 2013). These are more frequently localized in the white matter, basal ganglia, thalamus and pons, with surrounding astrocytes and oligodendrocytes (Chui, 2001; Moretti et al., 2005). Clinical characteristic features of sVAD are progressive signs of a dysexecutive syndrome, reduced planning, and cognitive flexibility, decreased processing speed, and behavior alterations, such as depression and apathy (Meyer et al., 2000; Moretti et al., 2011; Roh and Lee, 2014; Shi and Wardlaw, 2016).

Risk factors have been debated in dementia, especially metabolic features, hormonal changes, and vitamin alterations; these aspects have been related to a molecular pathogenesis in all the forms of cognitive decline, and hopefully, to a more effective management. Therefore, the target of many investigations has been to identify nutrition and lifestyle-based risk factors related to dementia, in order to develop possible future primary prevention efforts (Karakis et al., 2016). Many studies have been devoted to the increase of homocysteine, due to low folate and vitamin B12 deficiency in cognitive altered process; these studies focused on homocysteine effects on endothelium and neuronal disruption acting as a promoter of neurodegenerative events (Nelson et al., 2016; Smith and Refsum, 2016), but results are quite confounding, sometimes puzzling, and unclear (Malouf and Grimley Evans, 2008; Hainsworth et al., 2016; Moretti et al., 2017).

In the recent years, an increasing evidence supports a role for the fat-soluble vitamin D in brain function and development (Holick, 2004; McGrath et al., 2004; Schlogl and Holick, 2014; Granic et al., 2015). Vitamin D deficiency has been related with AD due to (1) a neurodegenerative accelerating property (Afzal et al., 2014; Prabhakar et al., 2015); (2) vascular acute damage, due to a presumed endothelium modification caused by a vitamin D direct effect (Sakurai et al., 2014; Prabhakar et al., 2015); and (3) to white matter hyper intensities (WMHs), lacunas and microbleeds in elderly with amnestic mild cognitive impairment (Sakurai et al., 2014; Chung et al., 2015). However, very few data are available regarding the vitamin D status in patients with pure sVAD (Prabhakar et al., 2015). Prabhakar et al. (2015) reported that hypertension and vitamin D deficiency considerably increase the odds of VaD (Prabhakar et al., 2015). On the other hand, a very recent study (Olsson et al., 2017) failed to show an association between baseline vitamin D-status and long-term risk of dementia or cognitive impairment over an 18 years period of time.

Therefore, the purposes of our cross-sectional study are to assess:

1. The differences in vascular risk factors and vitamin D-OH25 levels among groups of sVAD, AD, and healthy controls.
2. The association of folate, B12, homocysteine, and vitamin D with sVAD/AD.

## Subjects' characteristics

We conducted a cross-sectional study in a neurological group of patients, affected by AD and sVAD and compared their results with a normal healthy age population. From June 1st 2012 to June 1st 2015 a total of 543 cases were included in the study. 87 men and women had, suffering from Alzheimer's Disease, according to NINDCS-ADRDA (McKhann et al., 1984) criteria and the DSM-V (Fifth Edition), and 456 patients suffering from subcortical vascular dementia, in accordance with the NINDS-AIREN criteria (Chui et al., 1992; Román et al., 1993, see data and literature in Olsson et al., 2017). AD subjects had to show on brain MRI the pattern of hippocampal atrophy, and of the temporoparietal and precuneus regions (Weinstein et al., 1993). sVaD was diagnosed when the CT/MRI scan showed moderate to severe ischemic white matter changes and at least one lacunar infarct (Erkinjuntti et al., 1987; Marshall et al., 2006). As well accepted by literature (see data in Kim et al., 2014) all the patients had severe white matter hyperintensities on MRI, defined as peri-ventricular white matter hyperintensities, localized around the lateral ventricles or white matter hyperintensities, within the deep white matter (see data in Fazekas et al., 1987; Cleutjens et al., 2017). Brain CT-scans or MRI images were assessed independently by the neurologist (RM), after the radiologist's opinion. Brain CT-scans or MRI images were available for all the 543 patients; 362 patients did MRI studies, 181 did CT scans; 298 patients did CT plus MRI. A neurologist (RM) revised all the imaging, employing the Blennow scale for CT scans (Blennow et al., 1991; Wallin and Blennow, 1991) and the Scheltens scale for MRI imaging (Scheltens et al., 1993) in accordance with parameters of recent literature (Kim et al., 2014). There was 93.8% inter-rater agreement for the independent assessment of the scans (kappa = 0.79). Patients were not included in the study if they showed signs of normal pressure hydrocephalus, previous brain tumors, and previous diagnosis of major cerebrovascular disease, white matter lesions, caused by different specific etiologies, such as multiple sclerosis, collagen vascular disease, and genetic forms of vascular dementia (such as CADASIL or CARASIL). Patients with previous major psychiatric illness (i.e., schizophrenia, bipolar disorders, psychosis, compulsive-obsessive disorders, etc.) or central nervous system disorders and alcoholism were excluded too.

Exclusion criteria were the absence of an informed caregiver, unavailability of neuro-radiological examination, and/or the assumption of psychotropic drugs within 2 months prior to the clinical assessment. Seven patients were excluded by the lack of a sufficiently informed caregiver and 18 because they had assumed psychotropic drugs during the 2 months prior to our assessment.

Our control group was composed by healthy subjects, relatives, or caregivers of the patients, with no history of cerebrovascular diseases or degenerative disorders, who voluntarily accepted to take part in the study, matched for age, gender and educational level.

Study subjects underwent a standardized baseline assessment that included a detailed history, a physical examination, laboratory tests, and psychiatric evaluations. The physical examination included cardiac and blood pressure examination, peripheral pulses, retinal vessel, electrocardiographic evaluation. All patients were followed-up with periodical neurological and neuropsychological examinations. The present study was conducted in accordance with the Declaration of Helsinki and with the Ethics Guidelines of the Committee of the University-Hospital of Trieste, which approved it, and written informed consent was obtained from all the participants or from their caregivers.

## Methods

Patients with AD were grouped in Group A while patients with sVAD were assigned to Group B; controls were Group C. The main outcomes of the study for the AD and sVAD patients were: (1) global performance, assessed using the Mini-Mental State Examination (Folstein et al., 1975); (2) Frontal Assessment Battery (FAB; Dubois et al., 2000); (3) global behavioral symptoms, assessed by the Neuropsychiatric Inventory, NPI (Cummings et al., 1994); and (4) the caregiver stress, assessed by the Relative Stress Scale, RSS (Kinney and Stephens, 1989). Demographic details were registered for all the study subjects. All the patients and controls attended the LABS tests at the Hospital service, in order to reduce potentially different lab methods or different value parameters.

Hypertension was defined as diastolic blood pressure (DBP) ≥ 90 mm Hg and/or systolic blood pressure (SBP) ≥ 140 mm Hg. Diabetes mellitus was defined as venous plasma glucose concentration of ≥ 120 mg/dl after an overnight fast (Cummings et al., 1994). Glycated Hemoglobin (HbA1c) results have been aligned to the assay used in the Diabetes Control and Complications Trial (DCCT), expressed as a percentage (DCCT-HbA1c); non-diabetic “normal” range being 4–5.6%; 5.5–6.5 high risk of diabetes; more than 6.6% as having diabetes (WHO, IDF, 2006; Nathan et al., 2008; Weykamp, 2013). Fasting venous blood samples were collected, centrifuged immediately and stored at −80°C for further laboratory analysis. Clinical laboratory measurements, including serum total cholesterol, triglycerides, and high-density lipoprotein (HDL) cholesterol, have been determined enzymatically and low-density lipoprotein (LDL) cholesterol was calculated using Friedewald's formula (Friedewald et al., 1972). Serum levels of 25(OH)D were measured using enzyme immuno-assay kits (DIAsource Immunoassay S.A. Belgium) and quality control materials provided by the manufacturer. As Prabhakar et al. (2015) we employed the National Osteoporosis Society (NOS; Aspray et al., 2014) and therefore, subjects were categorized into Vitamin D deficiency [25(OH)D: ≤ 12 ng/ml], insufficiency [25(OH)D: 12–20 ng/ml] and sufficiency [25(OH)D: N 20 ng/ml] groups.

In order to have a complete evaluation of calcium metabolism, we tested at the same time the level of calcium and PTH. The level of folate, vitamin B12 levels and Homocysteine were also tested to obtain specific measures of vascular risk factors.

### Statistical analysis

Statistical analysis was performed with SPSS statistics 17.0 (SPSS, version 17.0). The difference in baseline characteristics between AD and sVAD and controls was assessed by ANOVA test for categorical variables; in case the ANOVA results were found significant, the multiple comparison analysis was also done by and the Tukey Test, to examine those two groups which were significantly different for each other. The multinomial logistic regression method was applied to analyze the relationship between disease status (AD, sVAD and control) considering them as dependent variable (non-metric) and age (metric), sex (non-metric), Hb1Ac, Cholesterol, and lipid parameters, calcium, PTH, vitamin D-OH25, folate, vitamin B12, and Homocysteine (all metric) as independent variable. The utility of present analysis (Multinomial Logistic regression) was assessed by classification accuracy, which compares the predicted disease group based on logistic model to the actual disease group (which is the value for dependent variable). Univariate odds ratios and 95% confidence intervals were estimated by binary logistic regression analysis. Spearmann's rank correlation analysis was calculated for the demographic variable. *P* ≤ 0.05 were considered statistically significant. Results are presented as mean with standard deviations, and *p*-values are presented where appropriate.

## Results

Eighty-seven AD patients and 456 sVAD patients were enrolled in the study. One AD patient and five sVAD patients died during the 12-months follow-up; therefore, 86 AD patients and 449 sVAD patients completed the study. Baseline neuropsychological characteristics of the study groups are presented in Table 1.

**Table 1:** Neuropsychological characteristics of study population (mean and SD in brackets).

**Characteristics**	**Group A**	**Group B**	**Group C**	***F* chi^2^ value**	**DF**	***p*-value**
Age	77.9 ± 2.01	75.65 ± 6.54	76.4 ± 2.3	2.66	2.24	0.73
MMSE score	19.4 (2.3)	24.2 (3.5)	27.9 (1.1)	0.71	2.157	0.01
FAB score	9.3 (2.5)	8.4 (1.3)	10.6 (1.2)	0.87	2.3	0.01
NPI score	20.3 (4.6)	16.1(3.2)	7.2 (3.1)	0.75	2.43	0.01
RSS score	42.3 (6.7)	27.3 (3.5)	8.1 (3.2)	0.89	2.02	0.01

The demographic variables i.e., age and gender were not significantly associated with the dementia status in both AD and sVAD. The demographic variables (age and gender) were not significantly associated with the dementia status in both AD and sVAD. One way analysis of variance (ANOVA) method was applied to explore the statistical significant difference among mean value in three groups (AD, sVAD, and control, Table 2). Four of the different biochemical variables (low folate and vitamin B12, low vitamin D-OH25, and high homocysteine) studied were significantly different (*p* < 0.001) in three groups (Table 2), which suggested that at least one average out of the three was statistically different than the other (Table 3); to explore such group, the multiple comparison analysis was done by Tukey test (Table 4). In AD group, mean vitamin D-OH 25 values (10.8 ± 1.05)were significantly lower than control (18.7 ± 3.05), mean folate levels (2.4 ± 0.3) were significantly lower than control (6.4 ± 0.2), mean vitamin B12 levels (129 ± 23.2) were significantly lower than controls (249 ± 13.2) and mean homocysteine levels were significantly higher (18.3 ± 3.5) than controls (11.1 ± 3.5).

**Table 2:** Comparison of mean value of age, gender, educational level, various biochemical parameters in AD, sVAD, and controls.

**Variable (Normal values)**	**Group A (86)**	**Group B (449)**	**Group C (567)**	***F* chi^2^ value**	**DF**	***p*-value**
Age	77.9 ± 2.01	75.65 ± 6.54	76.4 ± 2.3	2.66	2.24	0.73
Gender M/F	41/45	193/256	197/370	0.79	2	0.68
Educational level (years)	7.7 ± 3.4	8.1 ± 1.22	8.0 ± 0.9	0.67	3.11	0.45
Hb1ac (%)	6.1 ± 0.3	6.7 ± 0.7	5.6 ± 0.4	0.79	2.31	0.79
Cholesterol (mg/dl)	197.4 ± 34.6	207.5 ± 21.3	186.4 ± 24.6	0.67	2.251	0.75
Trygliceridis (mg/DL)	139.5 ± 23.9	129.3 ± 25.6	99.5 ± 34.2	0.87	2.13	0.67
HDL (mg/dl)	35.7 ± 12.1	31.1 ± 7.2	39.1 ± 14.1	0.67	2.45	0.43
LDL (mg/dl)	133.8 ± 13.4	150.5 ± 12.2	127.4 ± 7.4	0.87	2.65	0.37
Calcium (8.610.5 mg/dl)	9.8 ± 1.1	10.1 ± 0.4	11.2 ± 2.5	0.65	2.31	0.43
PTH (12–72 pg/ml)	39 ± 2.3	41.3 ± 2.7	37 ± 1.6	0.76	2.11	0.34
Vitamin D-OH 25 (30–100 ng/ml)	10.8 ± 1.05	9.1 ± 1.09	18.9 ± 3.05	2.66	2.251	0.01
Folate (3.89–26.0 ng/ml)	2.4 ± 0.3	1.9 ± 0.5	6.4 ± 0.2	0.71	2.157	0.01
Vitamin B12 (205–870 pg/ml)	129 ± 23.2	111 ± 26.2	249 ± 13.2	2.09	2.35	0.04
Homocysteine (3–15 mcmol/l)	18.3 ± 3.5	23.5 ± 3.4	11.1 ± 3.5	5.77	2.41	0.01

**Table 3:** Comparison of mean value of vitamin D-OH, folate, vitamin B12, and homocysteine (divided by different levels) parameters in D, sVAD, and controls.

	**Group A (86)**	**Group B (449)**	**Group C (567)**	***F* chi^2^ value**	**DF**	***P*-value**
Vitamin D-OH25 deficiency (≤ 12 ng/ml)	12 (14%) pts	49 (11%) pts	1 (0.17%) pts			
	8.1 (2.9) ng/ml	4.5 (2.1) ng/ml	11.1 (1.1) ng/ml	0.67	2.25	0.01
Vitamin D-OH25 insufficiency (12-20 ng/ml)	68 (79%) pts	387 (86%) pts	134 (24%) pts			
	15.7 (2.1)	12.1 (9.33)	19.1 (2.1)	0.87	2.13	0.01
Vitamin D-OH25 sufficiency (≥20 ng/ml)	6(7%) pts	13 (3%) pts	432 (76%) pts			
	23 (2.7)	21 (1.9)	29.4 (2.6)	0.71	2.6	0.43
Homocysteine (3-15 mcmol/l)	10 (11%) pts	23 (5.1%) pts	481 (84.8%) pts			
	10.7 (2.9)	11.1 (1.3)	8.4 (2.9)	0.81	2.1	0.47
Homocysteine (16–20 mcmol/l)	63 (74.8%) pts	301 (67.1%) pts	86 (15.1%) pts			
	18.5 (2.1)	19.3 (1.7)	17.1 (2.1)	0.84	2.6	0.05
Homocysteine (21–30 mcmol/L)	13 (15.1 %) pts	125 (21.8%) pts	0 pts			
	24.3 (2.1)	26.7 (2.5)	0	0.8	2.1	0.01
Folate (3.89–26 ng/ml)	6 (7%) pts	39 (8.7%) pts	503 (88.7%) pts			
	9.7 (1.1)	8.5 (2.3)	9.1 (1.7)	0.67	2.11	0.47
Folate (2–3.88 ng/ml)	78 (90.7%) pts	187 (41.7%) pts	63 (11.1%) pts			
	2.9 (1.2)	1.7 (2.3)	3.1 (1.0)	0.81	2.27	0.01
Folate (0.5–2.0 ng/ml)	2 (2.3%) pts	223 (49.6%) pts	1 (0.2%) pts			
	1.1 (20.7)	0.8 (1.1)	1.2 80.3)	0.81	2.1	0.01
Vit. B12 (205–870 PG/ML)	10 (11.6%) pts	132 (29.3%) pts	420 (74.1%) pts			
	264 (12.5)	251 (13.1)	305 (23.1)	0.86	2.6	0.45
Vit. B12 (100–204 PG/ML)	76 (88.4%) pts	317 (70.7%) pts	147 (25.9%) pts			
	125.4 (11.3)	102.6 (23.1)	167.4 (23.1)	0.59	2.6	0.05
Vit. B12 (50–99 PG/ML)	0 pts	0 pts	0 pts			
	0	0	0	na	na	na

**Table 4:** Multiple comparison analysis (Tukey test) of various biochemical parameters in AD, sVAD, and controls.

**Variable**	**Mean Diff**.	**SE of mean Diff**.	***p*-value**
**VIT. D-OH-25**			
A vs. C	−7.9	−2.01	0.01
B vs. C	−9.6	−1.96	0.01
**FOLATE**			
A vs. C	−4	−0.1	0.01
B vs. C	−4.5	−0.3	0.01
**VIT. B12**			
A vs. C	−120	−10.1	0.01
B vs. C	−138	−13-0	0.01
**HOMOCYSTEINE**			
A vs. C	+7.2	0.4	0.01
B vs. C	+12.4	0.1	0.01

In sVAD group, mean vitamin D-OH 25 values (9.1 ± 1.09) were significantly lower than control (18.7 ± 3.05), mean folate levels (1.9 ± 0.5) were significantly lower than control (6.4 ± 0.2), mean vitamin B12 levels (111 ± 26.2) were significantly lower than controls (249 ± 13.2) and mean homocysteine levels were significantly higher (23.5 ± 3.4) than controls (11.1 ± 3.5).

According to the NOS criteria (Aspray et al., 2014) we have found that there is a very significant difference in the prevalence of vitamin D-OH 25 deficiency and insufficiency between the three groups (Table 3). In AD population, 14% is deficient of vitamin D-OH25, 79% is insufficient, and only 7% is sufficient; in sVAD population, 11% is deficient of vitamin D-OH25, 86% is insufficient and only 3% is sufficient; whereas, in a healthy old population, even considering the declared cases of osteoporotic patients, only 0.17% is deficient of vitamin D-OH25, 24% is insufficient and 76% is sufficient. According to our laboratory cut-off scores, we have found that there is very significant difference in the prevalence of high values of homocysteine (16–20 mcmol/L) and very high levels of homocysteine (21–30 mcmol/L) between the three groups (Table 3). In AD population, 74.8% has high levels of homocysteine, 15.1% has very high levels of it and 11% is in normal range; in sVAD population, 67.1% has high levels of homocysteine, 21.8 % has very high values, and only 5.1% is in normal range; whereas in a healthy old population, only 15.1% has high levels of homocysteine, 84.8% is in normal range and 0% has very high levels of it. Considering our laboratory cut-off scores, we have found that there is a very significant difference in the prevalence of low levels of folate (2–3.88 ng/ml) and very low levels of folate (0.5–2.0 ng/ml) between the three groups (Table 3). In AD population, 90.7% has low levels of folate, 2.3% has very low levels of it and 7% is in normal range; in sVAD population, 47.1% has low levels of folate, 49.6% has very low values of it, and only 8.7% is in normal range; whereas, in a healthy old population, only 11.1% has low levels of folate, 0.2% has very low levels of it, and 88.7% is in normal range. Considering our laboratory cut-off scores, we also have found that there is a very significant difference in the prevalence of low levels of vitamin B12 (100–204 pg/ml) and very low levels of vitamin B12 (50–99 ng/ml) between the three groups (Table 3). In AD population, 88.4% has low levels of vitamin B12 and 11.6% is in normal range; in sVAD population, 70.7% has low levels of vitamin B12 and 29.3% is in normal range; whereas, in a healthy old population, only 25.9% has low levels of vitamin B12, and 74.1% is in normal range. Nobody reported very low levels of vitamin B12.

The univariate regression analysis reveals crude odds ratio for the association between AD and vitamin D insufficiency of 3.4 (95% CI: 10.93–13.56), *p* = 0.05 and vitamin deficiency of 5.6 (95% CI: 8.93–11.56), *p* = 0.023; there is an odd ratio for the association between AD and low levels of folate of 3.7 (95% CI: 2.1–3.4), *p* = 0.047 and very low levels of folate of 4.9 (95% CI: 1.1–2.1), *p* = 0.027; there is a odds ratio for the association between AD and low levels of vitamin B12 of 3.1 (95% CI: 112.1–203.4), *p* = 0.046; there is odds ratio for the association between AD and high levels of homocysteine of 4.5 (95% CI: 18.1–19.4), *p* = 0.036 and of 5.9 with very high levels of homocysteine (95% CI: 20.1–23.4), *p* = 0.016.

The univariate regression analysis reveals crude odds ratio for the association between sVAD and vitamin D insufficiency of 4.1 (95% CI: 11. 3–12.5), *p* = 0.034 and vitamin deficiency of 6.7 (95% CI: 7.3–9.6), *p* = 0.01; there is an odd ratio for the association between sVAD and low levels of folate of 4.1 (95% CI: 2.0–2.9), *p* = 0.03and very low levels of folate of 5.9 (95% CI: 1.1–2.1), *p* = 0.01; there is a odds ratio for the association between sVAD and low levels of vitamin B12 of 3.9 (95% CI: 101.1–163.4), *p* = 0.041; there is odds ratio for the association between sVAD and high levels of homocysteine of 5.9 (95% CI: 17.1–19.7), *p* = 0.01 and of 6.1 with very high levels of homocysteine (95% CI: 23.1–33.4), *p* = 0.016.

Table 5 shows the relationship of the disease state (AD and sVAD) with age, gender, vitamin D-OH25, folate, vitamin B12, and homocysteine levels. The presence of a relationship between them was checked based on the statistical significance of the final model chi-square and existence of a relationship was established. Out of the six considered independent variables, vitamin D-OH 25 levels, folate, and homocysteine had significant contribution toward AD and sVAD groups. Moreover, the regression coefficient (B) for vitamin D-OH25 was −0.46 for AD, indicating that the increase of vitamin D-OH 25 decreased the likelihood of dementia in AD group, with an exponential B value of 0.82 (95% CI: 2.5–10.11), which implies that for an increase of vitamin D-OH 25 levels the odds of having AD decreased by 18%; the regression coefficient (B) for folate was −0.88 for AD, indicating that the increase of folate decreased the likelihood of dementia in AD group, with an exponential B value of 0.91 (95% CI: 2.1–9.1), which implies that for an increase of folate levels the odds of having AD decreased by 9%; the regression coefficient (B) for homocysteine was + 0.57 for AD, indicating that the decrease of homocysteine decreased the likelihood of dementia in AD group, with an exponential B value of 0.85 (95% CI: 2.1–9.1), which implies that for a decrease of homocysteine levels the odds of having AD decreased by 15%.

**Table 5:** Summary of multinomial logistic regression analysis.

**Variable**		**Univariate association**			**Regression coefficient (B)**	**SE**	***p*-value**	**EXP (B)**	**95% CI for exponential (B)**
		**OR**	**95% CI**	***p*-value**					
**Dependent**	**Independent**								
AD	Age	1.1	1.1–2.3	0.76	0.023	0.015	0.6	1.006	0.9–1.03
	Sex	1.3	1.1–2.1	0.67	0.066	0.28	0.9	1.1	0.5–0.93
	Vit-D-OH-25	4.5	4.5–7.1	0.023	−0.46	0.12	0.01	0.82	2.5–10.11
	Folate	4.2	2.1–5.7	0.05	−0.98	0.17	0.01	0.91	2.1–9.1
	B12	1.8	1.2–4-5	0.45	−0.97	0.65	0.7	0.98	1.3–4.1
	Homocysteine	5.2	2.1–3.4	0.01	+0.57	0.12	0.01	0.85	2.7–10.4
sVAD	Age	1.3	1.4–2.7	0.81	0.016	0.022	0.45	0.98	0.94–1.07
	Sex	1.2	1.6–2.2	0.61	0.297	0.43	0.37	1.2	0.57–0.93
	Vit-D-OH-25	5.7	2.5–6.1	0.01	−0.39	0.12	0.01	0.79	2.0–17.1
	Folate	5.2	2.1–5.9	0.01	−0.77	0.23	0.01	0.89	2.1–8.7
	B12	2.1	1.2–7.1	0.074	−0.67	0.02	0.8	0.7	1.2–4.7
	Homocysteine	5.9	1.9–8.4	0.01	+0.23	0.01	0.01	0.8	2.1–13.4

The regression coefficient (B) for vitamin D-OH25 was −0.39 for sVAD, indicating that the increase of vitamin D-OH 25 decreased the likelihood of dementia in AD group, with an exponential B value of 0.79 (95% CI: 2.0–17.1), which implies that for an increase of vitamin D-OH 25 levels the odds of having sVAD decreased by 21%; the regression coefficient (B) for folate was −0.77 for sVAD, indicating that the increase of folate decreased the likelihood of dementia in sVAD group, with an exponential B value of 0.89 (95% CI: 2.1–8.7), which implies that for an increase of folate levels the odds of having sVAD decreased by 11%; the regression coefficient (B) for homocysteine was +0.23 for sVAD, indicating that the decrease of homocysteine decreased the likelihood of dementia in sVAD group, with an exponential B value of 0.8 (95% CI: 2.1–13.4), which implies that for an increase of homocysteine levels the odds of having sVAD decreased by 20%.

In present analysis, the classification accuracy rate of logistic model was 57.1% which was greater than the proportional by chance accuracy; the criteria for classification accuracy was satisfied.

In addition, serum 25(OH)D levels were found to be significantly and directly correlated with low levels of folate in the vitamin D-deficient group (Spearmann's correlation coefficient, *r* = 0.7 and 0.9, *p* < 0.01, respectively for Group A and B), and there is significant correlation between low levels of vitamin B12 in the vitamin deficient group (Spearmann's correlation coefficient, *r* = 0.8 and 0.9, *p* < 0.01, respectively for Group A and B), and finally, there is significant correlation with higher levels of homocysteine in the vitamin deficient and in the vitamin insufficient groups (Spearmann's correlation coefficient, *r* = 0.75 and 0.89, *p* < 0.01, respectively for Group A and B in the deficiency; Spearmann's correlation coefficient, *r* = 0.69, *p* < 0.05 and 0.71, *p* < 0.05, respectively for Group A and B in the insufficient).

## Discussion

This study was aimed to answer the questions specified in the introduction and namely whether alteration on the serum levels of folate, B12, homocysteine, and vitamin D may be related to neurodegenerative or vascular damage and our results are in line with previously reported studies (Friedewald et al., 1972; Folstein et al., 1975; McKhann et al., 1984; Erkinjuntti et al., 1987; Fazekas et al., 1987; Kinney and Stephens, 1989; Blennow et al., 1991; Wallin and Blennow, 1991; Chui et al., 1992; Román et al., 1993; Scheltens et al., 1993; Weinstein et al., 1993; Cummings et al., 1994; Dubois et al., 2000; Marshall et al., 2006; Moretti et al., 2006; WHO, IDF, 2006; Nathan et al., 2008; Weykamp, 2013; Aspray et al., 2014; Kim et al., 2014; Sakurai et al., 2014; Chung et al., 2015; Prabhakar et al., 2015; Cleutjens et al., 2017; Olsson et al., 2017).

We found that both A and B groups have lower folate and vitamin B12 levels and a higher homocysteine, even if more pronounced in sVAD than in AD. Of notice was the data obtained with vitamin D-OH 25 level: we have established that 93% of our AD patients and 97% of our sVAD patients suffered either from deficiency or from insufficiency of vitamin D-OH 25. Moreover, we found that for an increase of vitamin D-OH 25 levels the odds of having AD decreased by 18%, for an increase of folate levels the odds of having AD decreased by 9% and for a decrease of homocysteine levels the odds of having AD decreased by 15%. In sVAD we found that for an increase of vitamin D-OH 25 levels the odds of having sVAD decreased by 21%, for an increase of folate levels the odds of having sVAD decreased by 11% and for an increase of homocysteine levels the odds of having sVAD decreased by 20%.

Our study has several limitations:

1. It is a single-center study
2. It has been designed as a cross-sectional study
3. The number of patients is small to interfere
4. It has no pathological confirm

It has some strengths:

1. All the patients can be fully examined
2. All the patients attended neuroimaging and neuropsychological evaluation
3. We have examined two distinct dementing conditions, mainly degenerative (AD) and vascular, related to small vessel disease pathologies (sVAD)
4. We have a healthy old control group
5. For the first time, we produced data concerning the superimposing effects of homocysteine high levels and low vitamin D-OH 25.

Our results seem to have some accordance with many other in literature (Afzal et al., 2014; Sakurai et al., 2014; Chung et al., 2015; Prabhakar et al., 2015) as far as vitamin D-OH low levels, but not with a recent one (Olsson et al., 2017). It also seems in accordance with many other as far as homocysteine and folate (see data and literature in McCully, 1969; Ueland et al., 2000; Hogervorst et al., 2002; Moretti et al., 2017) but not with many other (Homocysteine Studies Collaboration, 2002; Obeid and Herrmann, 2006; Miles et al., 2016).

The novelty of our work is that we report the parallel effect of two controversial factors: homocysteine and vitamin D in two different dementing condition, AD and sVAD, and it seems that the two variables create a detrimental effect, which seems to promote the neural pathology, more evident in vascular condition.

How can vitamin D-OH and homocysteine influence the neural system? Do they act as neurodegenerative promoters or as pure vascular damage factors?

Although homocysteine and vitamin D-OH 25 are different from biochemical and structural properties, their activity is surprisingly similar. The accumulation of homocysteine and the deficiency of vitamin D-OH are both toxic to neuronal cells. They might affect neuronal plasticity (Streck et al., 2003), with different mechanisms in early or in adult life, and improve neurodegeneration (Obeid and Herrmann, 2006), alter brain energy productions mechanisms (Streck et al., 2003), potentiate inflammation (Lazarewicz et al., 2003; Herrmann et al., 2006) and reduce the endothelium response to oxidation processes (Lazarewicz et al., 2003; Streck et al., 2003; Herrmann et al., 2006). Homocysteine regulates calcium inflow, via the activation of group I metabotropic glutamate receptors (Lipton et al., 1997; Lazarewicz et al., 2003; Robert et al., 2005; Herrmann et al., 2006; Obeid and Herrmann, 2006), and this has a relevance in the induction of brain lipid peroxidation process, and expanding the neural calcium-related apoptosis mechanism (Blom and Smulders, 2011). Moreover, homocysteine accumulation has an amyloidogenic effect by inducing the endoplasmic reticulum protein HERP, which potentiates the c-secretase activity and enhances the accumulation of AB1-40 in the brain (Mok et al., 2002; Seshadri et al., 2002). Homocysteine also increases the neural vulnerability to the damage created by amyloid accumulation (Morris, 2003). Hyper-homocystenemia upregulates PS1 genes, promoting a hypomethylation of PPM1 (Leulliot et al., 2004), causing, therefore, a hyperphosphorilation of tau protein, which seems to potentiate the microtubules transport mechanism. The activation of caspase-3 by hyperhomocystenemia promotes the accumulation of amyloid on smooth muscle cells of small vessel disease (Leulliot et al., 2004; Chun et al., 2016), which leads to a dysregulation of cerebral blood flow, commonly observed in AD and in vascular dementia. Higher homocysteine levels can be considered as an independent risk factor for moderate to severe leukaraiosis in patients with AD (Pushpakumar et al., 2014; Zhou et al., 2014). Moreover, homocysteine metabolism is regulated by the redox potential in the cell (Blom and Smulders, 2011; Pushpakumar et al., 2014; Zhou et al., 2014), by disruption of the trans-sulfuration pathway (Seshadri et al., 2002; Obeid and Herrmann, 2006; Blom and Smulders, 2011). Homocysteine is a precursor of hydrogen sulfide (H2S), which revealed to be a potent vasodilator and it regulates the vessel diameter, it seems to protect the endothelium from redox stress and chronic inflammation (Pushpakumar et al., 2014; Zhou et al., 2014). The accumulation of homocysteine can cause a reduction in nitric oxide synthesis and potentiate oxidative endothelium stress (Pushpakumar et al., 2014; Zhou et al., 2014).

When we consider vitamin D, the results are surprisingly similar. Animal models demonstrated alterations in fetal and adult animal models induced by a deficiency of vitamin D-OH 25. Vitamin D modulates different processes, such as neurogenesis, cell proliferation, differentiation, and neurotransmitter metabolism (Eyles et al., 2013, 2014; Cui et al., 2015). By exposing fetal animals to vitamin D deficiency, an evident alteration of dopamine and NMDA circuitries can be observed, with clear consequences for altered behavior, memory and motor assessment (Becker et al., 2005; Turner et al., 2013; Eyles et al., 2014; Cui et al., 2015; Overeem et al., 2016). On the other hand, the exposition of previously normally developed brains to vitamin D deficiency seems to result in memory impairment, with major conduct alterations and with less executive possibilities (Byrne et al., 2013). These brains have a reduction of the glutamic acid decarboxylase (key enzymes in gamma-aminobutyric acid (GABAergic inter-neurons), and show decreased levels of glutamate and glutamine in brain tissue (Byrne et al., 2013). In a key study (Brewer et al., 2001), it was found that vitamin D protected rat primary hippocampal cultures from excitotoxicity insults (i.e., glycine and NMDA). Patch clamp studies found that, L-type voltage-dependent calcium currents were reduced following incubation with vitamin D (Brewer et al., 2001; Carlberg et al., 2005; Balden et al., 2012; Byrne et al., 2013; Gezen-Ak et al., 2013; Suzuki et al., 2013; Cui et al., 2015); therefore it has been hypothesized that vitamin D-OH 25 might influence calcium inflow, and consequently it can regulate dendritic cell functions and neural apoptosis (Carlberg et al., 2005; Gezen-Ak et al., 2013; Suzuki et al., 2013; Cui et al., 2015). On the other hand, vitamin D deficiency has been related to altered myocardial functions, associated with a ventricular dilation and impaired electromechanical coupling (O'Connell et al., 1997; Xiang et al., 2005; Simpson, 2011). Much more stimulating is the endothelial cells expression of the receptors for vitamin D; these receptors are up-regulated under inflammation condition in endothelial cells (Xiang et al., 2005; Bodyak et al., 2007; Wong et al., 2008) and vitamin D analogs decrease endothelium adhesion molecules, and protect against advanced glycation products (Young et al., 2011), reduce vascular smooth muscle contractions and vascular tone in hypertensive models, modulating the calcium influx across endothelial cells (Manolagas et al., 1986; Danielsson et al., 1996; Somjen et al., 2005; Wong et al., 2008; Oh et al., 2009).

However, it cannot be denied that results in Literature, concerning the two above-mentioned biochemical variables, are wide-ranging, controversial and sometimes even contradictory.

In accordance with modern and recent approaches in Literature, neuro-inflammation is the possible point in common between the two factors. We support this idea and speculate on the fact that low levels of vitamin D and high levels of homocysteine, in co-existence, might lead to an altered response to inflammation, and therefore predispose to microvascular and endothelium damages. Different reports documented the Th1 induced homocysteine inflammation response (Murr et al., 2001), and it appears that higher levels of homocysteine can be detected in chronic inflammatory conditions, even if vitamin B12 and folate are in range. Many studies documented that higher levels of homocysteine are related to an increment of neopterin and Il-6 (Bleie et al., 2007), which can be partially modulated only by a correct implementation of folate. Thus, it has been suggested that “an optimal folate status over-ride the influence of immunostimulation on Th1 by Hcy” (Bleie et al., 2007). Li et al. (2015) showed that an animal model induced hyper-Hcy produced higher plasma levels of tumor necrosis factor alpha (TNF-α) and Interleukin 1 beta (IL-1β) and therefore induced a trigger of inflammation. These results have been confirmed by many other reports (Yi-Deng et al., 2007; Krishna et al., 2013; Zhou et al., 2014) which showed that higher levels of Hcy promotes, in many different experimental conditions, the activity of specific but different genes, implicates in methylation process, and caused inflammation of the endothelium matrix and atherosclerosis (Yi-Deng et al., 2007; Krishna et al., 2013; Zhou et al., 2014). In different chronic medical conditions, like Rheumatoid Arthritis (Essouma and Noubiap, 2015) higher levels of homocysteine are more frequent than in the general population, and that hyper-homocysteine in RA creates a chronic condition of oxidative stress, prothrombotic induction, and, indirectly, by the excess of ROS released, it up-regulates the Nuclear Factor Kappa B, considered as one of “the master regulator of the expression of inflammatory genes” (Essouma and Noubiap, 2015; Ying et al., 2015). A very recent work has demonstrated that cytokines released by microglia can activate NF-KB signaling resulting in an enhanced expression of the pro-inflammatory system, such as Toll-like receptors (TLR), in particular, TLR2, 4 and 9 (Zhou et al., 2016). They are hyper-expressed by microglia and directly activate NF-KB in “*in vitro*-BV2 model” of PD (Zhou et al., 2016). Different new studies pointed out the topic of inflammation as a strong basis of many different neurodegenerative pathologies, like PD, where *in vitro* specific models, like 6-OHDA- lesioned PC12 cells highly expressed COX2, IL-2 and TNF-alpha (Rong et al., 2003; Xu et al., 2013). In the same *in-vitro* PD model, it has been established the inflammatory mediator participation of a nuclear receptor subfamily, the so-called NUR, involved as a transcriptional factors, in all the process involving neuronal development and in the response to inflammatory attacks (Rong et al., 2003). In particular, Nur 77 acts as a potent modulator of the macrophage and T cell response (as pointed out by Wei et al., 2016), and indirectly promotes inflammation and mitochondrial dysfunction and therefore causes apoptosis. In particular, there is a well-documented increase of the cytosolic level of Nur 77, with a well-described translocation from the nucleus to the cytosol, following the oxidative stress in the PD *in vitro*-model (Gao et al., 2016; Wei et al., 2016). Even in the vascular dementia model, inflammation is claimed as a determinant factor: different biomarkers have been claimed to be over-expressed, such as CysC (Jonsdottir et al., 2013), which seems to be increased in AD and in VAD patients, compared to healthy subjects, and correlates with the severity of the disease (Chen et al., 2015). In the same line, many other biochemical variables, such as HDL, have found to be increased in plasma in AD and in VAD patients. HDL probably promotes an anti-oxidative stress response in damaged brain structures (Wen et al., 2017). Even uric acid acts as a natural anti-oxidative stress factor (acting as a possible disease modifier in MSA and PD) (Jin et al., 2010; Yilmaz and Granger, 2010). It has been inflammatory process plays a dominant role in the pathogenesis of brain ischemia and contributes to stoke formation (Broughton B. R. et al., 2013). Anoxia induces the production of reactive oxygen species and an induction of ICAM1, VCAMS, Selectins, integrins on endothelium leukocytes and platelets (Broughton B. R. S. et al., 2013; Azizieh et al., 2016).

In the same line go all the most recent studies on vitamin D-OH25 deficiency, in order to promote inflammation and endothelium degeneration (Mangin et al., 2014; Na et al., 2014).

When considering all these results, it can be strongly supported the hypothesis of combined and shared roles of vitamin D and homocysteine in neurodegeneration and in vascular and endothelium disruption.

## Conclusions

The questions which we made at the very beginning have been finally answered.

Many doubts remain; how do homocysteine and vitamin D act to create damage? Do they potentiate neurodegeneration or microvascular alteration?

We have observed that low levels of vitamin D are more present in dementia populations, in degenerative and in small-vessel types; these two groups share hyperhomocystenemia, too; the combined presence of both factors is significantly higher in these two groups. More studies will be needed to implement further knowledge.

## Author contributions

RM designed the study and is the responsible of the data. PC and CC analyzed the data. SG, MD, and CT revised the paper, contribute to its written parts and to the analysis of Literature.

### Conflict of interest statement

The authors declare that the research was conducted in the absence of any commercial or financial relationships that could be construed as a potential conflict of interest.
